# Comparison of structures and inhibition activities of serine protease inhibitors of *Trichinella spiralis* and *Trichinella pseudospiralis*

**DOI:** 10.1186/s13578-025-01375-0

**Published:** 2025-03-13

**Authors:** Ruixue Li, Bing Zhang, Chen Chen

**Affiliations:** Department of Biochemistry and Molecular Biology, School of Basic Medical Sciences, Tianjin Medical University, Tianjin, 300070 China

**Keywords:** *Trichinella spiralis*, *Trichinella pseudospiralis*, Serine protease inhibitor, Crystal structure

## Abstract

**Background:**

Trichinosis is one of the most widespread parasitic infections worldwide. *Trichinella spiralis* not only infects humans but can also utilize wild anddomestic animals as hosts. The serine protease inhibitors secreted by *Trichinella spiralis* play a critical role in its invasion and immune evasion. Serpins can effectively inhibit host proteases, although the host can mount a strongimmune response against to these inhibitors.

**Results:**

In this study we analyzed the crystal structures of the serine protease inhibitors from *Trichinella spiralis* and *Trichinella pseudospiralis*, revealing that both serpins exhibit.structural characteristics typical of serine protease inhibitors. The similarity of both “breach” region and “shutter” region of the two serpins are very high, but the “hinge” region are different, the “hinge” of Tp-serpin is closed, while of Ts-serpin was partially inserted into sheet-A, suggesting that Tp-serpin had higher inhibition activity. Using alpha chymotrypsin as Ts-serpin and Tp-serpin protease targets, the two serpins enzyme inhibition activity were measured separately, by measuring the secondary inhibition rate constant, half inhibitory concentration IC50, inhibition of stoichiometric number parameters and confirmed both the serine protease inhibitory activity, and Tp-serpin slightly higher than that of Ts-serpin, but no inhibition activity of P1-P1’ mutant.

**Conclusion:**

In this study, the mechanism of enzyme inhibition activity of serpin was studied by means of structural biology and biochemistry comprehensively. These discoveries provide a theoretical foundation for a deeper understanding of the inhibition mechanisms of serpins and for the development of new drugs and vaccines against *Trichinella spiralis* infection.

**Supplementary Information:**

The online version contains supplementary material available at 10.1186/s13578-025-01375-0.

## Background

Trichinellosis is a serious worldwide food-borne zoonotic disease [1], primarily prevalent in developing countries, with *Trichinella spiralis* being the major pathogen forthis disease [2]. Trichinella spiralis not only infects humans, but also completes its life cycle in wild and domestic animals [3]. The main clinical symptoms include fever, headache, abdominal pain, diarrhea, orbital or facial edema, myalgia, and in servere cases, death due to heart complications [4]. Researchers have identified 12 species and genotypes, which can be classified into two main evolutionary clades, the encapsulated species *Trichinella spiralis* (*Ts*) and the non-encapsulated species *Trichinella pseudospiralis* (*Tp*) [3]. To ensure long-term survive within the host, both *Trichinella* species must evade the host’s proteolytic enzymes, particularly serine proteases, which are involved in digestion and immune responses. This evasion is a critical mechanism for the parasite to avoid host defenses [5]. Serine protease inhibitors (serpins) secreted by *Trichinella spiralis* play a significant role in host-parasite interactions by regulating host digestive serine proteases such as trypsin and chymotrypsin [6]. Therefore, studying the structure and function of *Trichinella spiralis* serpins is of great importance.

Serpins representthe largest superfamily of protease inhibitors [7]. They play a crucial role in regulating numerous vitalprocesses and maintaining homeostasis in mammals. In case of serpinopathies, gene mutations lead to the production of inactive serpins or the formation of protein aggregates that lose their functio [8]. Serpins achieve inhibition through conformational changes and the kinetic trapping of enzyme intermediates.Specifically, they exhibit high specificity by binding the exposed reactive center loop (RCL) and exosites to the active site on the target protease [9]. A comprehensive understanding of the structure and function of serpins can offer new insights for the design of engineered protease inhibitors for specific therapeutic applications.

I this study we focused on the serpins derived from *Trichinella spiralis* and *Trichinella pseudospiralis*. We resolvd the three-dimensional structures of the serpins from *Trichinella spiralis* (Ts-serpin) and *Trichinella pseudospiralis* (Tp-serpin), analyzed their active conformations, and identified key elements within their native structures. We explored the similarities and differences between these inhibitors and proposed potential mechanism of protease inhibition based on proteins from the same family. Using enzymatic reaction kinetics, the study investigated the kinetic parameters of Ts-serpin and Tp-serpin, determined the type of inhibition exhibited by these protease inhibitors, and confirmed the specific location of the active region through mutational analysis. Additionally, the enzymatic properties of serpins as inhibitors were evaluated, highlighting their inhibitory effects. This work elucidated the biological significance of serpins, natural molecules from the parasitic genus *Trichinella*, from both structural and biochemical perspectives. Tick serpins were inoculated into Kunming mice for immune protection analysis. The results demonstrated that these serpins exhibited anticoagulant functions and could inhibit the activity of CD4 + lymphocytes. This indicates that tick serpins possess significant immune protection potential and could be developed into a vaccine [10]. Furthermore, vaccination of rabbits with recombinant tick serpin suggested that serpins could serve as potential candidates for a cocktail anti-tick vaccine [11]. Similarly, serpins from Trichinella also show promise as candidates for vaccine development, and the structural analysis of Trichinella serpins could reveal antigenic surface epitopes. Additionally, compound 3034 (1,3-benzoxazole-6-carboxylic acid) was found to directly bind to serpinB9 and inhibit its interaction with granzyme B [12], suggesting that inhibitors targeting the binding between serpins and host proteases could be screened based on serpin structures, contributing to drug development. The findings underscored the potential of serpins as drug targets and provide a foundation for the exploring potential antiparasitic vaccines. Furthermore, engineering serpins may restore the function of dysfunctional proteases, which are implicated in various diseases, offering a promising avenue for the development of new therapeutic strategies.

## Methods

### Protein expression and purification

The Ts-serpin gene was cloned into expression vector pGEX-6P-1 which attaches an N-terminal glutathione S-transferase (GST) tag and a preScission protease cleavage site and The Tp-serpin gene was cloned into expression vector pET-28b. The recombinant proteins were expressed in *E. coli* BL21(DE3) (Sangon Biotech) respectively. The bacteria were cultured in LB medium containing 100 µg/mL ampicillin or kanamycin at 37℃. Recombinant proteins expression was induced with 0.5 mM isopropyl-1-thio-β-D-galactopyranoside.

The cells of recombinant protein Ts-serpin were collected and resuspended in PBS buffer (10 mM Na_2_HPO_4_, 1.8 mM KH_2_PO_4_, 137 mM NaCl, 2.7 mM KCl, pH 7.4) and disrupted using ultrasonication. Centrifugation at 18,000 rpm for 40 min. The supernatant was incubated with GST-Tag Protein purification Kit(Agarose Resin) (YEASEN, 0753ES10), using PBS buffer (NCM Biotech, N1014) to remove the unbound proteins. PreScission protease was incubated with protein for overnight at 4℃, and the target protein contained in flow-through was collected and concentrated, then purified by ion-exchange chromatography and gel filtration chromatography. Finally, the target proteins were replaced in the storage buffer (50 mM Tris-HCl, 200 mM NaCl, pH 8.0) and concentrated to 6 mg/mL, 12 mg/mL. Small aliquots were flash frozen and stored at -80 °C for future use.

The supernatant of recombinant proteins Tp-serpin were incubated with Anti-His magnetic beads (Life-iLab, AP62L202), using wash buffer (50 mM Tris-HCl, 500 mM NaCl, 20 mM Imidazole, pH 8.0) was used to remove the unbound proteins. Finally, the target proteins were collected and concentrated same with above.

### Crystallization, data collection and processing

Ts-serpin and Tp-serpin crystallized by sitting drop at 16℃. Crystals were grown by mixing equal volumes of 6 mg/mL and 12 mg/mL protein solution (1 µL) with the reservoir solution (1 µL). The crystallization condition of Ts-serpin containing 32% polyethylene glycol 8 K, 0.15 M ammonium sulfate, 0.2 M sodium cacodylate trihydrate, pH 6.8. The crystallization condition of Tp-serpin containing 22% polyethylene glycol 8 K, 0.2 M sodium chloride, 0.1 M sodium acetate trihydrate, pH 4.0, 30% w/v D-(+)-Glucose monohydrate. To prevent the crystal from being damaged by liquid nitrogen, the crystal was soaked with crystallization solution containing 20% (v/v) glycerol and then rapidly freezed in liquid nitrogen. The diffraction data were recorded in Shanghai Synchrotron Radiation Facility (SSRF) Beamline 19U and Beamline 17U1. Final data statistics was shown in Table 1.

**Table 1:** Data collection and refinement statistics

Structure	Ts-serpin	Tp-serpin
PDB	9J88	9J91
Data collection		
Wavelength (Å)	0.97918	0.97913
Space group	*P* 2_1_ 2_1_ 2_1_	*P* 6_2_ 2 2
Resolution range(Å)	29.62–2.34 (2.43–2.34)^*^	27.76–2.60 (2.69–2.60)^*^
Unit cell a, b, c (Å)	60.54, 71.62, 143.69	175.49, 175.49, 63.42
α, β, γ (°)	90, 90, 90	90.00, 90.00, 120.00
Unique reflections	26,731 (2449)	18,284 (1772)
Completeness (%)	99.05 (92.63)	99.80 (99.55)
Redundancy	9.5(9.3)	19.8(18.7)
*I/σ(I)*	18.3 (2.6)	20.0 (2.7)
*R*_merge_(%)	13.5(79.3)	13.8(164.1)
*CC*_*1/2*_	0.99 (0.92)	0.99 (0.70)
Refinement		
Reflections used in refinement	26,728 (2450)	18,282 (1772)
Reflections used for *R*_free_	1345 (116)	883 (81)
*R*_work_ (%)	19.0	20.7
*R*_free_ (%)	25.8	24.3
Protein residues	688	343
Water	268	268
Number of non-hydrogen	5755	2844
Protein	5502	2705
Solvent	253	139
RMSD values		
Bond lengths (Å)	0.008	0.009
Bond angles (°)	1.27	1.06
Ramachandran favored (%)	96.57	93.73
Ramachandran allowed (%)	3.28	6.27

*Numbers in the brackets represent the highest resolution shell

The structure of Ts-serpin was determined by molecular replacement using regions of mutant serpinB1 structure [13] (4GA7) as the searching model. The structure of Tp-serpin was determined by molecular replacement using the regions of conserpin structure [14](5CDX) as the searching model. The models building was done by Coot and refinement by Phenix [15, 16]. Figures were prepared using PyMOL [17].

### Enzyme activity inhibition assay of serpin and its mutants

Mixtures containing 3 nM of chymotrypsin and increasing concentrations (0 nM, 10 nM, 20 nM, 40 nM, 80 nM, 160 nM, and 320 nM) of Ts-serpin, Tp-serpin, Ts-serpin-Mut, and Tp-serpin-Mut were prepared in 96-well microtiter plates. Incubated at 25 °C for 15 min, then added 150 µM of substrate BTEE. Set up 3 replicates for each group, mixed well, and incubated at 25 °C for 1 h. Measured the absorbance at 256 nm, and set the untreated wells as 100% protease activity. The inhibition rate of serpin against chymotrypsin was calculated using the following formula: enzyme activity inhibition rate = (OD value of untreated wells - OD value of experimental wells) / OD value of untreated wells.

### Determination of α-chymotrypsin inhibition kinetics by serpin

The serpin can covalently bind to α-chymotrypsin, its inhibition type is irreversible inhibition, and its inhibition effect needs to be characterized by the inhibition constants *K*_*i*_ and *k*_*3*_. When determining its inhibition constant, the apparent first-order inhibition rate constant *k*_*obs*_ should be fitted by formula 4.4.1. Where *P* represents the absorbance value of the product generated by the substrate reaction, *v*_*0*_ represents the initial reaction rate, *t* represents the reaction time, and *D* represents the absorbance of the reaction initiation system. According to Formula 4.4.2, the inhibition constants *K*_*i*_ and *k*_*3*_ can be calculated by linear fitting of *k*_*obs*_ with different inhibitor concentrations. Where *[I]* is the concentration of inhibitor, *[S]* is the concentration of substrate, *K*_*m*_ is the Michaelis constant obtained above, *K*_*i*_ represents the equilibrium constant of irreversible binding of inhibitor and enzyme, and *k*_*3*_ represents the reaction rate constant during the reaction of inhibitor.

4.4.1 $$P = \left( {{{{v_0}} \mathord{\left/{\vphantom {{{v_0}} {{k_{obs}}}}} \right.\kern-\nulldelimiterspace} {{k_{obs}}}}} \right)\left( {1 - \exp \left( { - {k_{obs}}t} \right)} \right) + D$$

4.4.2 $$\frac{1}{{{k_{obs}}}} = \frac{1}{{{k_3}}} + \frac{{{K_i}}}{{{k_3}}}\left( {1 + {{\left[ S \right]} \mathord{\left/{\vphantom {{\left[ S \right]} {Km}}} \right.\kern-\nulldelimiterspace} {Km}}} \right) \cdot \frac{1}{{\left[ I \right]}}$$

Mixtures containing 3 nM of chymotrypsin and increasing concentrations (0 nM, 50 nM, 75 nM, 100 nM, 125 nM, 150 nM, 200 nM) of serpin were prepared in 96-well microtiter plates. Incubated at 25 °C for 15 min, then added 150 µM of substrate BTEE. Set up three replicates for each group, mixed well, and incubated at 25 °C. Measured the absorbance at 256 nm. The reaction time was taken as the horizontal coordinate and the absorbance value was taken as the vertical coordinate to fit the line, and the slope was obtained as the *k*_*obs*_ value, that is, the first-order association rate constant. Three replicates were set up for each group. Then, with the serpin concentration as the horizontal coordinate and the *k*_*obs*_ value obtained above as the ordinate, the inhibition constants *K*_*i*_ and *k*_*3*_ were obtained.

### Determination of IC50 inhibition of α-chymotrypsin by serpin

Mixtures containing 3 nM of chymotrypsin and increasing concentrations (0nM, 10nM, 20nM, 40nM, 80nM, 160nM, 320nM and 640nM) of serpin were prepared in 96-well microtiter plates. Incubated at 25 °C for 15 min, then added 150 µM of substrate BTEE. Set up three replicates for each group, mixed well, and incubated at 25 °C. Measured the absorbance at 256 nm and set the untreated wells as 100% protease activity. After adding inhibitor, through the calculation formula, the inhibition rate of enzyme activity = (OD value of the group without serpin - OD value of the experimental group)/OD value of the group without serpin. The inhibitory rate of serpin on chymotrypsin was obtained. With the concentration as the horizontal coordinate and the inhibition rate as the vertical coordinate, the logarithmic function curve was fitted to obtain the half suppression rate (IC50) of serpin.

### Inhibition stoichiometric (SI) measurement of serpin

Stoichiometry of inhibition (SI) represents the number of inhibitors required for 1 mol of protease to be inhibited. serpin with different concentrations (0-30nM) and chymotrypsin with a final concentration of 30nM were mixed, incubated at 25℃ for 15 min, then added 100 µM of substrate BTEE. Set up three replicates for each group, mixed well, and incubated at 25 °C. Measured the absorbance at 256 nm. The remaining activity of chymotrypsin was calculated with the relative activity of enzyme (%) as the vertical axis and the molar ratio of serpin to chymotrypsin ([I]/[E]) as the horizontal axis, where ([I]/[E]) were 0.125, 0.25, 0.325, 0.5, 0.75, 1.0, 1.5, 3.0, respectively. The line is fitted and the SI value is based on the linear regression equation, that is, the intersection of the line and the horizontal axis.

### Temperature-dependent inhibitory assays

Mixtures containing 3 nM of chymotrypsin and 30nM serpin were prepared in 96-well microtiter plates. Incubated at different temperatures (25℃, 30℃, 37℃, 42℃ and 50℃) for 15 min, then added 100 µM of substrate BTEE. Set up three replicates for each group, mixed well, and incubated at 25 °C. Measured the absorbance at 256 nm. According to the calculation formula, the relative enzyme activity = 1- (OD value of the group without serpin - OD value of the experimental group)/OD value of the group without serpin. Calculate the relative enzyme activity at different temperatures and plot the curve.

### pH-dependent inhibitory assays

Mixtures containing 3 nM of chymotrypsin which diluted with different pH buffers (pH 2.0, 4.0, 6.0, 8.0, 10.0, 12.0) and 30nM serpin were prepared in 96-well microtiter plates. Incubated at 25 °C for 15 min, then added 100 µM of substrate BTEE, substrate and serpin were diluted with the same group of pH buffer. Set up three replicates for each group, mixed well, and incubated at 25 °C. Measured the absorbance at 256 nm. Relative enzyme activity = 1- (OD value of the group without serpin - OD value of the experimental group)/OD value of the group without serpin, calculate relative enzyme activity under different pH conditions and draw a curve.

## Results

### Structure of Ts-serpin and Tp-serpin

The structure of the Tp-serpin (amino acids 3-377) was resolved by molecular replacement based on data collected in space group P6_2_ 2 2 at 2.60 Å resolution. The structure of the Ts-serpin (amino acids 2-373) was resolved by molecular replacement based on data collected in space group P2_1_2_1_2_1_ at 2.34 Å resolution. The data collection and refinement statistics was shown in Table 1.

**Fig. 1:**
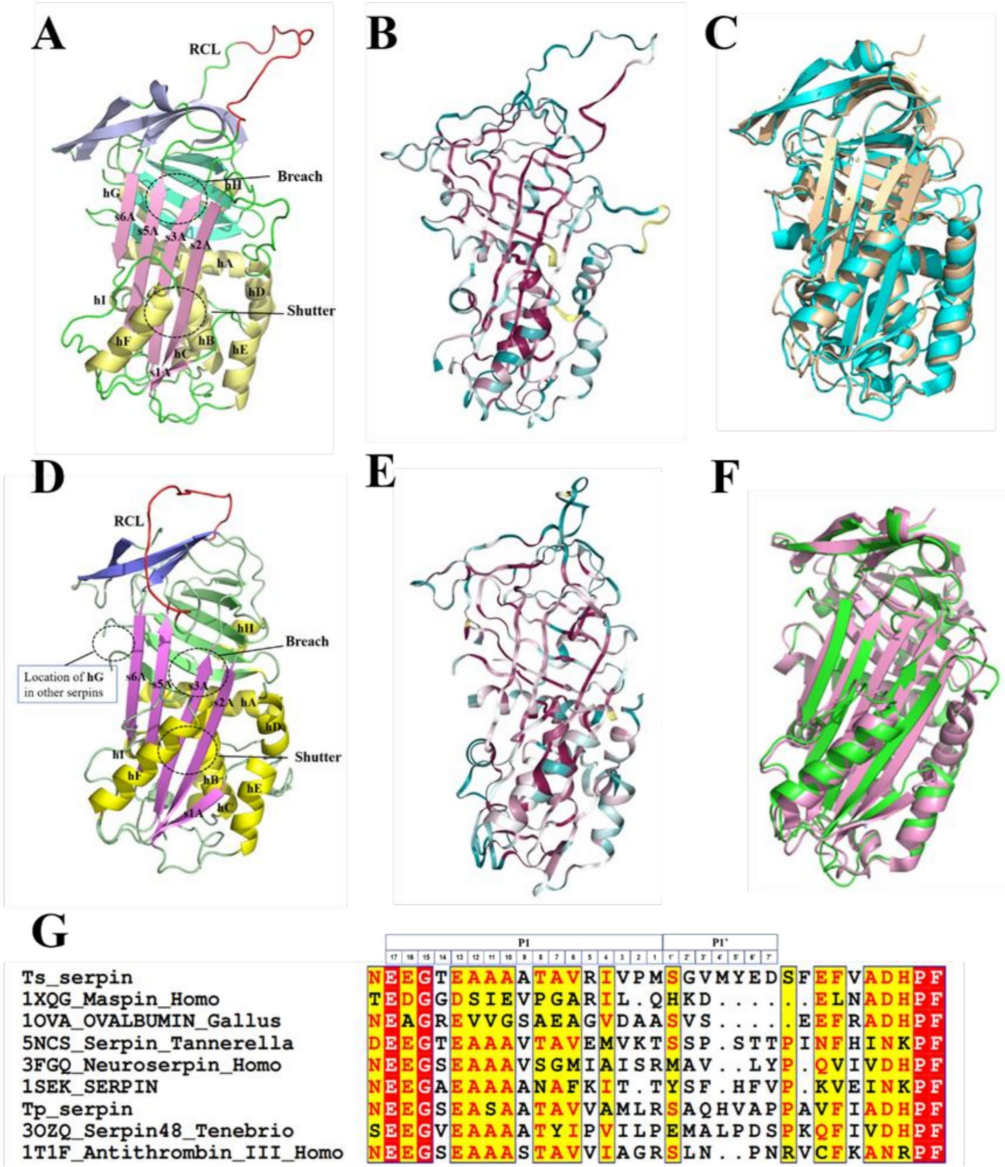
Overall structural of Tp-serpin and Ts-serpin. (**A**) Ribbon model of the Tp-serpin. Sheets A-C are shown in light pink, light purple and light green. RCL (in red), which is in the structure there was no electron density at the corresponding position; The helical are in light yellow; The flexible regions are in turquoise. The Breach and the shutter area are highlighted in dotted elliptical boxes. (**B**) Structure conservation of Tp-serpin. (**C**) Comparsion of structure of Tp-serpin in golden with conserpin (PDB:5CNX) [18] in cyan. (**D**) Ribbon model of the Ts-serpin. Sheets A-C are shown in mauve, purple and green. RCL (in red), which is in the structure there was no electron density at the corresponding position; The helical are in yellow; The flexible regions are in livid gray. The Breach and the shutter area are highlighted in dotted elliptical boxes. (**E**) Structure conservation of Ts-serpin. (**F**) Comparsion of structure of Ts-serpin in green with Human SerpinB3 (PDB:4ZK0) [19] in pink. (**G**) Alignment of RCL sequences of Ts-serpin and Tp-serpin with other serpins

Tp-serpin is a monomer characterized by an overall globular fold composed of three vertical, partially overlapping, twisted three antiparallel β-sheets (termed sA, sB, and sC) surrounded by a cluster of nine α-helices (hA-hI) and RCL. The A β-sheet contains five β-strands (Fig. 1A). The RCL in red is fully expelled from the A β-sheet, no electron density was observed in this region, this portion of the molecule being disordered and only the first two residues of the RCL can be seen protruding from the molecule at the top of strand s5A. The six-stranded B β-sheet comprises the majority of the hydrophobic core of the molecule, and the four-stranded C β-sheet includes strand s1C located at the C-terminal end of the RCL (Fig. 1A). The Loop area is shown in cyan. The end of the A β-sheet is the *N* end of the Tp-serpin, while the end of the s4B of the B β-sheet is the C-terminal. The positions of the Breach and Shutter areas are marked with dotted boxes.

However, the structure of the Ts-serpin with two Ts-serpin molecules in each crystallographic asymmetric unit. Three antiparallel β-sheets surrounded by a cluster of eight α-helices hA-hF and hH-hI. The G-helix, a feature of all eukaryote serpin structures solved to date, is absent in Ts-serpin (Fig. 1D). In contrast to Tp-serpin, Ts-serpin contains an extended C-terminal sequence that interacts with the top of the A β-sheet (Fig. 1D). Through structural conservation analysis of Ts-serpin and Tp-serpin, it was found that both have typical serpin structural characteristics (Fig. 1B and E).

In order to explore the functions of Tp-serpin and Ts-serpin, this paper conducted sequence similarity and structural similarity analysis on Tp-serpin and Ts-serpin. By searching in the PDB database, it was found that the protein with the highest sequence similarity (39.6%) to Tp-serpin was an artificially designed protein called Conserpin [20], in which the C-terminus of RCL was truncated to make it more stable (Fig. 1C). It is speculated from the above that Tp-serpin may have a good folding state in terms of structure and dynamic properties during the evolution process to adapt to the complex digestive enzyme environment of the host. The eukaryotic serpin with higher sequence similarity to Tp-serpin is human Neuroserpin [21], reaching 37.8% (Fig. S1A). The similarity with human leukocyte elastase inhibitor (SERPINB1) [22] is up to 32.8% (Fig. S1B). Based on the functions of the above similar protease inhibitors reported in the literature, it is speculated that Ts-serpin may interact with host-specific serine proteases after entering the host, and exert its physiological activity through its suicidal conformational change during the inhibition process. According to the structural similarity analysis of Tp-serpin, the results are shown in the Table S1. Most of the structures similar to Tp-serpin are important protease inhibitors in the human. By comparing the sequences of these structurally similar proteins, it can be seen that the serpins of different species have lower sequence similarity (Fig. S2), indicating that serpins still maintain good structural conservation in the evolutionary relationship, but have different inhibitory characteristics when they exerted their activity. The three-dimensional structure of Maspin (PDB: 1WZ9) analyzed by Karl Volz’s team [23] has the characteristics of serpin folding, but the RCL of Maspin is unique in length, composition, and location, so it has anti-tumor.

properties. The structure of Maspin is similar to that of Tp-serpin (RMSD = 1.6 Å) (Fig. S1C), but the RCL portion of Maspin reduced seven flexible amino acids compared with Tp-serpin, which makes it more stable. It is speculated that Tp-serpin can be modified and developed as a drug to inhibit tumor.

Ts-serpin had a high similarity with Pso p27, a protein related to the pathogenesis of psoriasis [19], with a similarity of 36.2%. Pso p27 is a highly antigenic protein produced in the mast cells of psoriatic plaques, and studies have shown that Ts-serpin also has strong antigenicity [24]. The structural analysis and discussion of its epitope will be of guiding significance for the development and preparation of trichinella vaccine. Structural similarity search showed (Table S2) that Antithrombin (PDB: 2BEH, RMSD = 1.6 Å) [25]and Antithrombin III (PDB: 2ANT, RMSD = 1.5) [26] had the highest similarity, followed by human heterochromatin associated protein MENT.

(PDB number: 2H4R, RMSD = 1.6 Å) [27] and Leukocyte elastase inhibitor (PDB: 4GA7, RMSD = 1.7 Å) (Fig. S1D, S1E, S1F).

### Analysis and comparison of key domains of Ts-serpin and Tp-serpin

The wild-type serpin has an exposed RCL that connects S5A and S1C and protrudes from the top of the molecule. It spans 22 residues (corresponding to positions P17-P5’) and does not interact with each other, suggesting that it may be flexible and disordered in solution, allowing it adapts to the active site cleavage of different target peptidases. The RCL is divided into a hinge region spanning P17-P9 (Fig. 1G), which is crucial for the RCL-induced conformational rearrangement, and an exposed loop region from P8 to P5’, which contains the theoretical cleavage site (P1-P1’) (Fig. 1G).

**Fig. 2:**
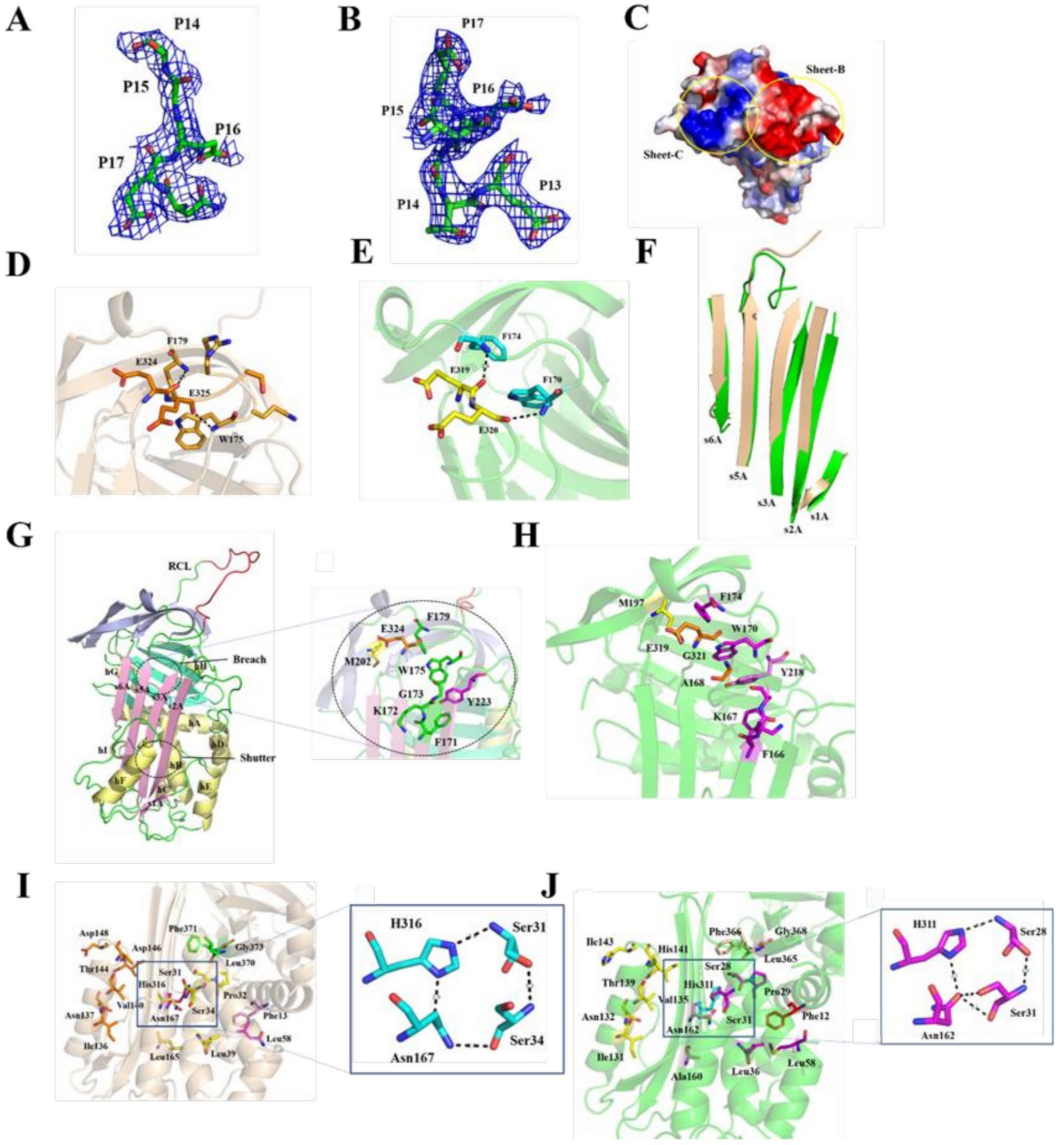
Structural characteristics of Tp-serpin and Ts-serpin. (**A**) The unbiased Fo-Fc electron density map for P17-P14 of Tp-serpin contoured at 1.0 sigma is shown as blue mesh. (**B**) The unbiased Fo-Fc electron density map for P17-P13 of Ts-serpin contoured at 1.0 sigma is shown as blue mesh. (**C**) Surface charge distribution of sheet-B and sheet-C of Ts-serpin. (**D**) Diagram of hydrogen bond interaction between RCL and s3A of Tp-serpin. (**E**) Diagram of hydrogen bond interaction between RCL and s3A of Ts-serpin. (**F**) Comparison diagram of sheet-A of Ts-serpin in green and Tp-serpin in golden. (**G**) The breach domain of Tp-serpin. The amino acids of s3A and loop-s3A-s4C in green, the amino acids of s3C in yellow, the amino acids of s2B in magenta, and the amino acids of the hinge region of the RCL in orange. (**H**) The breach domain of Ts-serpin. The amino acids of s3A and loop-s3A-s4C in fushcia, the amino acids of s3C in yellow, the amino acids of s2B in pink, and the amino acids of the hinge region of the RCL in orange. (**I**) The shutter domain of Tp-serpin. The amino acids of hA in pink, the amino acids of loop-s6B-hB and hB in yellow, the amino acids of hC in magenta, the amino acids of hF in orange, the amino acids of s3A in poloere, the amino acids of s5A in vermilion, the amino acids of s5B and C-terminal in green. (**J**) The shutter domain of Ts-serpin. The amino acids of hA in red, the amino acids of loop-s6B-hB and hB in green, the amino acids of hC in purple, the amino acids of hH in yellow, the amino acids of s3A in pink, the amino acids of s5A in cyan, the amino acids of s5B and C-terminal in golden

The RCL of Tp-serpin is composed of residues Glu324 (P17) to Val345 (P5’). The entire RCL is only clearly visible in the electron density map at P17-P14 (Fig. 2A). Tp-serpin is similar to typical serine protease inhibitors, with the typical conformation of the RCL in the active center, suggesting that the RCL can bind to the active region of the target proteinase. Similar to Tp-serpin, the RCL of Ts-serpin has a clear density of P17-P13 (Fig. 2B). However, unlike Tp-serpin, the sheet-B and sheet-C near the RCL in Ts-serpin have strong electricity (Fig. 2C), presumably to prevent the RCL from interacting with its substrate.

The residues P17-P9 of the N-terminal of the RCL belong to the hinge region of the Tp-serpin, located in the surface groove formed by the corner ts3AhF1 (defining the turn after s3A and before the spiral F1 as ts3AhF1). The base of the groove is formed by the conserved residue Trp (W175) in the serpin. The hinge can form a complex hydrogen bond interaction network with ts3AhF1. Through sequence comparison, it was found that the glycine of P15 was extremely conserved, which facilitated the occurrence of folding corners. There is also a glycine at P15 of the Tp-serpin, causing it to form a hydrogen bond between Glu325O-Trp175N and Phe179N-Glu324O (Fig. 2D).This interaction is also present in Ovalbumin [28] and Antichymosin [29]. The residues P17-P13 of the N-terminal part of RCL are the hinge region of Ts-serpin, which is similar to the Tp-serpin hinge region, and can form a complex hydrogen bond interaction network with ts3AhF1. P15 is a conserved glycine, which is convenient for folding corners to form hydrogen bonds between Glu320O-Trp170N and Phe174N-Glu319O (Fig. 2E). Unlike Tp-serpin, the hinge region of Ts-serpin is inserted between s3A and s5A, similar to the intermediate state between the natural active state of serpin and the cut inactivated state, so that the length of RCL exposed to the solvent is shortened, the flexibility is reduced, and the binding domain with the substrate protease is reduced. Therefore, the inhibitory activity of Ts-serpin may be lower than that of Tp-serpin.

The Tp-serpin obtained in this paper is a natural metastable structure without s4A. The hydrogen bond between s3A and s5A is analyzed, and it is found that a tight hydrogen bond network is formed between s3A and s5A of β sheet-A. L165 of s3A via hydrogen bonds, and a single water molecule creates a bridge between N167 of s3A and F315 of s5A (Fig. S3A). According to the folding characteristics of serpin, when RCL tries to insert into sheet-A, the hydrogen bond formed between s5A and s3A will be broken, which facilitates the insertion of s4A chain after RCL is cut and the formation of a complex. Like Tp - serpin, Ts-serpin forms a hydrogen bond network between s3A and s5A. Unlike Tsp-serpin, three water molecules bridge the Y314 and K312 of s5A with F166 and I164 of s3A (Fig. S3B).

### Analysis of breach and shutter domains

Two regions have been proven to be critical to regulating conformational change and inhibitory activity of serpins. First, the breach is located at the initial insertion point of the chain at the top of the A β-sheet. The Tp-serpin breach include residues Phe171, Lys172, Gly173, Trp175 and Phe179 of s3A and Loop-s3A-s4C; Met202 of s3C; Tyr223 of s2B, Glu324 and Gly326 of the RCL hinge region (Fig. 2G). Similar to Tp-serpin, Ts-serpin breach include residues Phe166, Lys167, Ala168, Trp170, and Phe174 of s3A and Loop-s3A-s4C; Met197 of s3C; Tyr218 of s2B, Glu319 and Gly321 of the RCL hinge region (Fig. 2H). Due to the insertion of the hinge region, the distance between s3A and s5A expands from 8.0 Å to 11.2 Å, and the side chain direction of key amino acids is shifted (Fig. 2F).

Second, the shutter is composed of a series of conserved amino acid clusters, located in the middle of the molecule, centered on the top of the B-helix, and is postulated to control opening of sheet-A and insertion of RCL. The Tp-serpin shutteras shown in Fig. 2I. Overall, the amino acid residues of shutter and breach constitute a relatively conserved set of sites that are necessary for core structural integrity and are 70% conserved in the inhibitory serpins. Among them, the hydrogen bond network formed by four amino acids in shutter region is highly conserved, mainly including His of s5A and Asn of s3A, and two Ser of helix B. This domain is a checkpoint in the RCL’s full insertion path that controls the opening and closing of the lower half of sheet-A. Mutations in these four amino acids of serpin in the human can lead to dysfunction and disease. It can be seen that these amino acids have important roles. The Ts-serpin shutter as shown in Fig. 2J. Compared with Tp-serpin, it has higher structural and sequence conservation.

### Analysis and comparison of natural conformational between Ts-serpin and Tp-serpin

When RCL is cleaved by protease hydrolysis, s4A is formed and inserted between s3A and s5A, which causes large conformational changes in s3A, s2A and helix D. The hinge region of the RCL of Tp-serpin (P17-P13) interacts with ts3AhF1 to maintain the active conformation of s3A, s2A and helix D. P16 interacts with Gly173 in s3A of Tp-serpin to directly stabilize the conformation of s3A. In addition, ts3AhF1, ts2Bs3B (a corner in sheet-B), and thDs2A (a corner behind helix D) form a large number of hydrogen bond interactions (Table S3). Therefore, RCL is directly involved in the stability of serpin’s natural conformation. Unlike Tp-serpin, P16 has a direct interaction with Tyr165 in Ts-serpin’s s3A, which stabilizes the conformation of s3A. In addition, similar to Tp-serpin, ts3AhF1, ts2Bs3B and thDs2A of Ts-serpin form a large number of hydrogen bond interactions (Table S4), and the number of hydrogen bonds and salt bridges are more than that of Tp-serpin, which is speculated to be more compact in structure, and its natural stability is higher because the RCL hinge region is partially inserted into sheet-A.

### Enzymatic inhibition of natural Ts-serpin and Tp-serpin and their mutants

By analyzing the three-dimensional structure of the Ts-serpin and Tp-serpin, it is speculated that they have serine protease inhibitory activity. Compared with the same family of proteins, it was found that its active site is mainly concentrated in the P1-P1′ part of RCL (Fig. 1G). Under the induction of proteases, Ts-serpin and Tp-serpin will hydrolyze the peptide bond between P1-P1′. Therefore, the inhibitory activities of natural Ts-serpin, Tp-serpin and their mutants were measured through enzyme activity assay methods to further study their specific inhibitory mechanisms. In order to verify that the P1 residue and P1′ residue in the RCL region are the specific key sites that determine the inhibitory mechanism, the P1 and P1′ amino acid residues in the RCL of the two serpins were mutated.

**Fig. 3:**
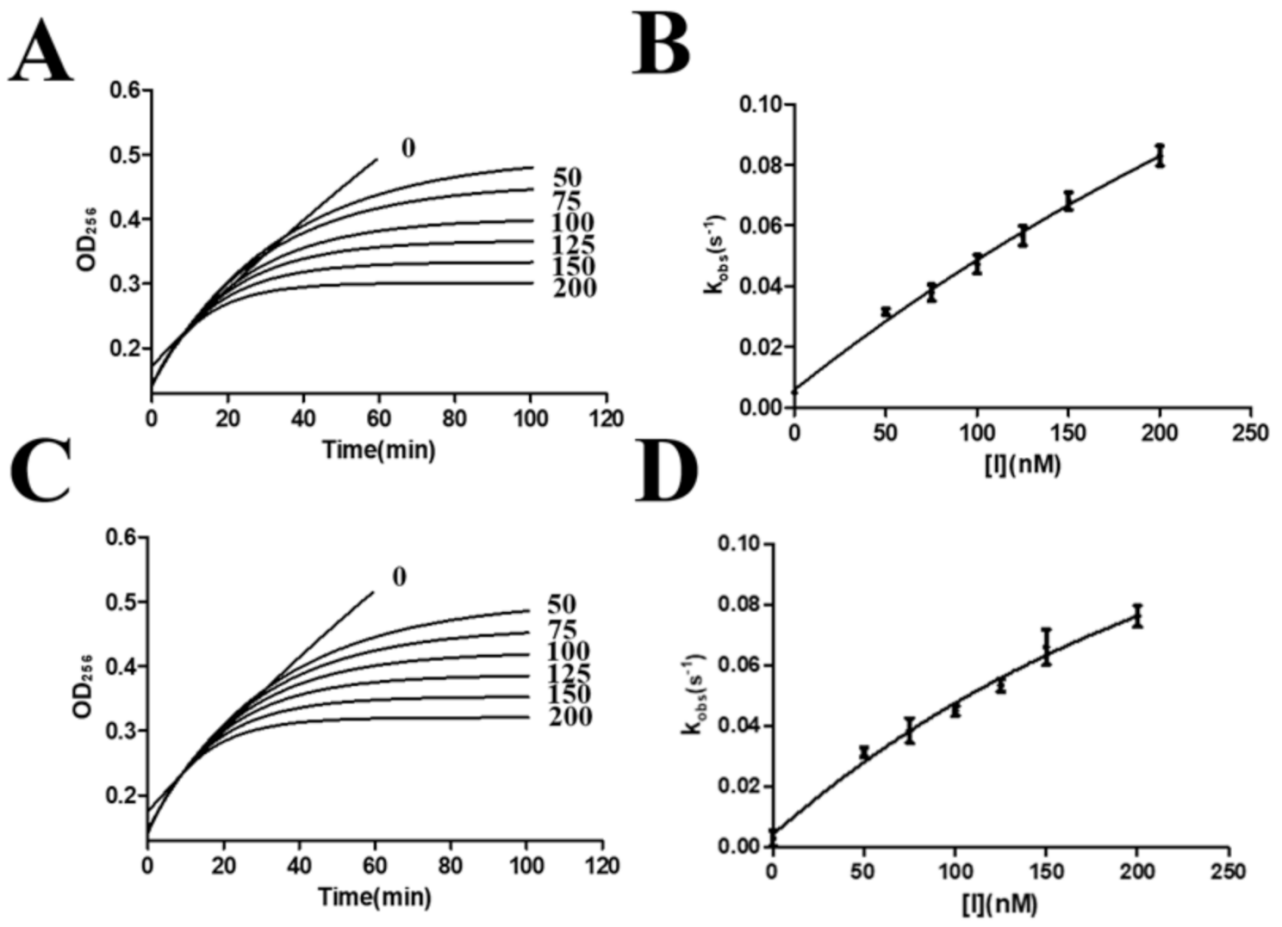
The inhibition curve of Tp-serpin and Ts-serpin to α-chymotrypsin. (**A C**) The fitting curves of enzymatic inhibition over time. (**B D**) Fitting function plot of Ts-serpin and Tp-serpin concentration versus *K*_*obs*_

First, the inhibitory activity of serpin on α-chymotrypsin was determined (Fig. 3). The inhibitory constants *K*_*i*_ and *k*_*3*_ of Tp-serpin on α-chymotrypsin were 47.92 ± 2.35 nM and 2.35 ± 0.06 (×10^− 3^s^− 1^) respectively, while Ts-serpin of 49.22 ± 3.39 nM and 2.47 ± 0.12 (×10^− 3^s^− 1^) (Table 2). It shows that both Tp-serpin and Ts-serpin have good inhibitory effects on α-chymotrypsin, and the *K*_*i*_ values ​​of the two enzyme inhibitors are relatively close, which indicates that their affinities for α-chymotrypsin are relatively close. The *k*_*3*_ values ​​of the two inhibitors are close, indicating that their inhibitory effects are equivalent. Tp-serpin has slightly higher inhibitory activity than Ts-serpin.

**Table 2:** The inhibiton constant Ot α-chymotrypsin inhibited by Ts-serpin and Tp-serpin

Name	*k*_3_(×10^− 3^s^− 1^)	*K*_*i*_(nM)
Ts-serpin	2.47 ± 0.12	49.22 ± 3.39
Tp-serpin	2.35 ± 0.06	47.92 ± 2.35

**Fig. 4:**
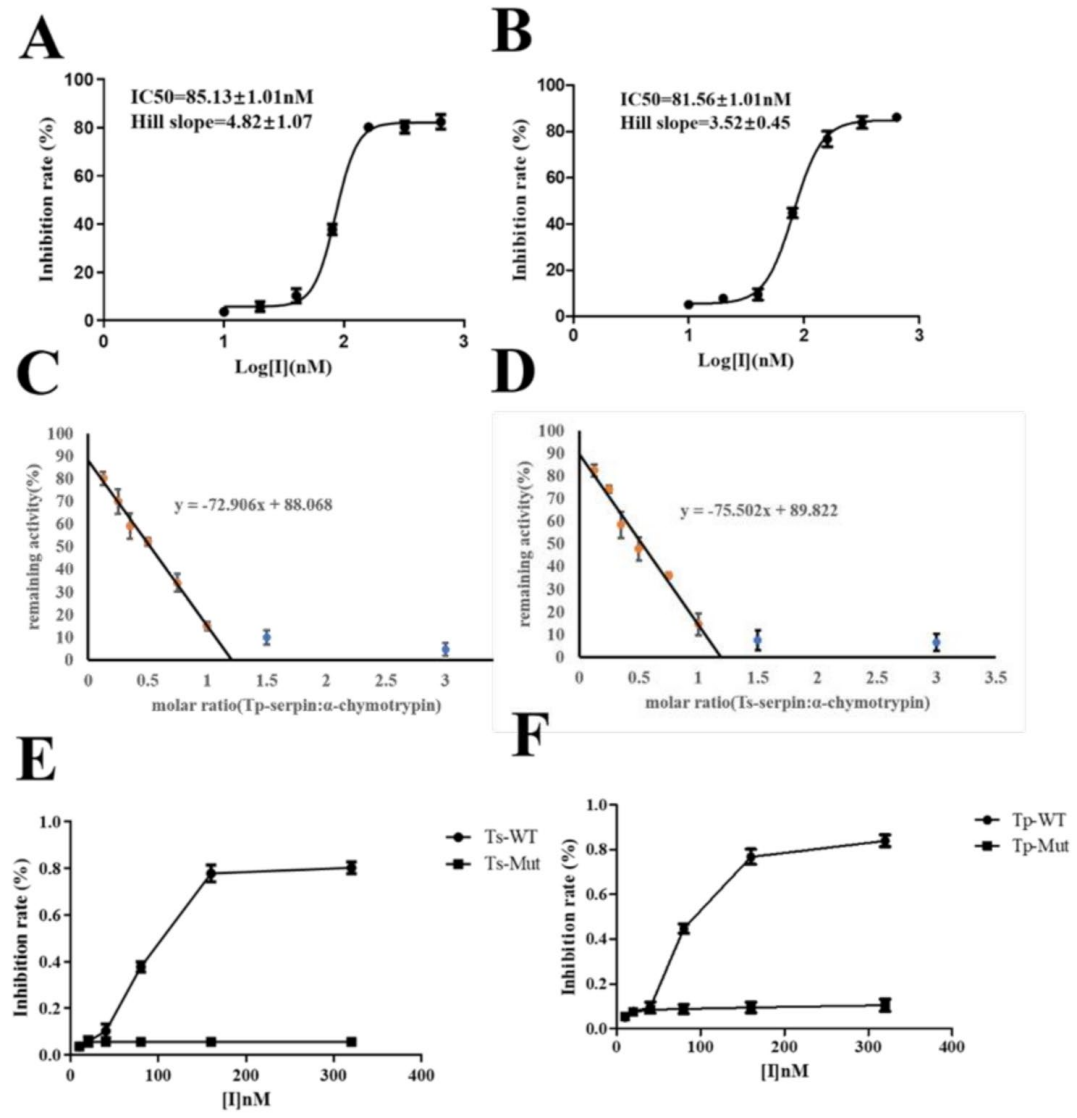
The inhibition activity of Serpin to α-chymotrypsin. (**A**) The IC50 inhibition of Ts-serpin to α-chymotrypsin. (**B**) The IC50 inhibition of Tp-serpin to α-chymotrypsin. (**C**) Stoichiometry of inhibition of Ts-serpin. (**D**) Stoichiometry of inhibition of Tp-serpin. (**E**) Comparison of inhibitory activities of Ts-serpin and its mutants. (**F**) Comparison of inhibitory activities of Ts-serpin and its mutants

Next, the experiment measured the inhibition rate of serpin on chymotrypsin. Taking the logarithmic concentration as the abscissa and the inhibition rate as the ordinate, fitting the logarithmic function curve through Graphpad prism 5.0 software, the half-inhibition rate (IC_50_) of Ts is 85.13nM (Fig. 4A), while the half-inhibition rate (IC_50_) of Tp is 81.56nM (Fig. 4B). The inhibitory stoichiometry (SI) of Serpin was also determined. The SI values ​​of Ts-serpin and Tp-serpin were 1.208 (Fig. 4C) and 1.189 (Fig. 4D) respectively.

Both serpin and protease are proteins, so temperature and pH have a certain impact on the biological activity and stability of these two molecules. By using different pH and temperature conditions, the inhibitory effects of Ts and Tp serpins on α-chymotrypsin enzyme activity showed that the environment of the enzymatic reaction has a great influence on the activity of enzymes and enzyme inhibitors. The results showed that both Tp-serpin and Ts-serpin had high serine protease inhibitory activity against α-chymotrypsin in a temperature range between 30 and 42 ◦C, the optimal reaction temperature of both serpins is 37 °C (Fig. S4A, S4B). However, when the temperature is higher than 42 °C, the activity of serpin will be greatly reduced, and even partially deactivated. In a pH range between 6.0 and10.0, both Tp-serpin and Ts-serpin have high serine protease inhibitory activity against α-chymotrypsin, with the optimal reaction pH being 8.0 (Fig. S4C, S4D).

**Fig. 5:**
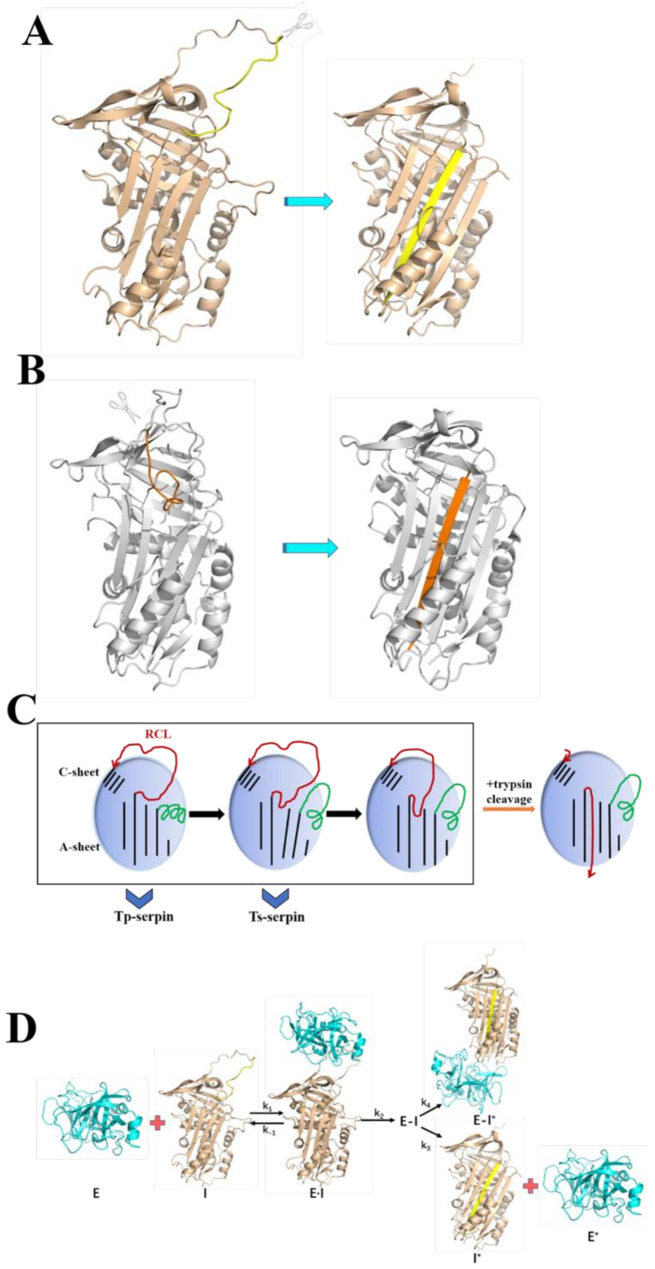
Prediction of conformational change of Ts-serpin and Tp-serpin. (**A**) The structure conformational change of Tp-serpin. (**B**) The structure conformational change of Ts-serpin. (**C**) There are two different states of Ts-serpin and Tp-serpin. (**D**) The mechanism of serpin-protease complex. Serpin [I] in gold and protease [E] in cyan combine to form the Michaelis complex[E·I]. This complex is acylated [E-I], resulting in RCL cutting and insertion into Sheet-A in yellow and pulling the protease closer to the end of s2A [E-I*]. Upon completion of the deacylase translocation of a portion of the acylase complex, the serpin and free protease in the cut state are released. K_1_=[E·I]/([E]*[I]), K_− 1_=([E]*[I])/[E·I]

Finally, the enzyme inhibitory activity of serpin and its mutants was measured. As shown in the Fig. 5, the inhibitory activity of 200nM Tp-serpin and Ts-serpin on α-chymotrypsin is more than 80%, which has good protease inhibitory activity, but the mutation P1-P1′ has a great impact on its inhibitory activity, significantly reducing its activity (Fig. 4E and F).

### Prediction of conformational change of Ts-serpin and Tp-serpin

Serpin, as a protease inhibitor, inactivates its target protease through a suicide substrate inhibition mechanism. The protease and serpin are specifically recognized to form an initial non-covalently binding complex (called the Michaelis complex), and the protease then cleats the RCL to form a covalent acylase intermediate. The serpin conformation changes significantly, resulting in a translocation of the protease about 70 Å from the initial reaction center ring docking site to the opposite direction of the serpin. The change in the active center involves the insertion of the hinge (P17-P13) portion near the RCL, forming a central sheet-A. In this paper, the metastable structures of two serpins are analyzed, and through the above analysis, it is speculated that models can be constructed from induced conformational changes (Fig. 5A and B). In Tp-serpin, the RCL of P3-P3 ‘is typical and thus able to interact with the target protease, and the hinge region of the RCL stabilizes its conformation through interaction with sheet-A. The RCL observed in Tp-serpin may represent the resting conformation, while the RCL of Ts-serpin may represent an intermediate in which sheet-A is unstable (Fig. 5C). In addition, in the model depicted in Fig. 5D, the rate-limiting step is an intermolecular kinetic process, while the intramolecular changes is negligible.

## Discussion

We have determined the x-ray crystal structure of the *Trichinella* serpin Ts-serpin and Tp-serpin in the native conformation. These two serpins adopt the typical native serpin fold, possessing a 5-stranded A β-sheet with the RCL fully expelled from the top of the A β-sheet. The peptide bond formed by the P1 residue and the P1′ residue of RCL is a cleavable bond. The target protease will recognize the cleavage site and hydrolyze it. The specificity of proteases for serpin is exerted through its RCL, and whether serpin family members are inhibitory also depends on the sequence and structure of RCL.

Serpins that normally have inhibitory activity undergo large conformational changes. When released from the Michaelis serpin-protease complex, the RCL of serpin is cleaved, and the N-terminal fragment of the cleaved bond is inserted into sheet-A as the s4A chain. The two broken ends are separated by a distance of 70Å. This large-scale rearrangement is driven by the greatly enhanced stability of the cleaved serpin, which results from the insertion of P3-P15 residues of RCL into sheet-A in an antiparallel long-chain conformation, for this is necessary for the inhibitory activity of the serpin-protease complex. By docking the complex of Tp-serpin and α-chymotrypsin (Fig. 5D), it can be observed that Tp has structural similarities with other serpins in the active site, and it is speculated that it can exert its activity as an effective serine protease inhibitor.

The crystal structure of Ts-serpin shows that the conformation of the N-terminal hinge region of RCL is subtly but important different from that of other serpins except Antithrombin. The N-terminus of RCL is partially inserted into sheet-A to form the fourth chain of sheet-A. A similar phenomenon can also be observed in the native conformation of Antithrombin [30]. Studies have shown that the reduced RCL length of the native conformation of Antithrombin is due to its partial insertion into sheet-A, which may also be related to its poor inhibitory activity against Coagulation active factors Xa and IXa in the absence of heparin [31, 32], so it is speculated Ts-serpin may be weaker than Tp-serpin in its ability to inhibit enzyme activity.

Next, the inhibitory abilities of two serpins, Ts-serpin and Tp-serpin, on α-chymotrypsin were determined. The results showed that the serpins of both parasites could inhibit its enzyme activity in a dose-dependent manner. Trichinella spiralis serpin inhibited α-chymotrypsin. Bovine α-chymotrypsin has high inhibitory activity, with inhibition rates as high as over 80%. It can be inferred that Trichinella spiralis and Pseudotrichinella spiralis can interact with the host intestinal tract extracellularly in the early stages of infection. Combined with digestive enzymes, it has an inhibitory effect on its protease activity and blocks the host’s immune response. At the same time, the epitope region of the protease inhibitor is bound by the protease, which reduces the immunogenicity and avoids the specific antibody response of the host in the early stage of infection, which has a positive effect on Trichinella spiralis and Pseudotrichinella spiralis evading host immune regulation.

## Conclusion

These findings highlight serpins as possible drug targets, and by studying their structures, key epitopes can be discovered, providing direct evidence for the development of potential antiparasitic vaccines. While engineering serpins can normalize dysfunctional proteases, which are at the root of many diseases, it shows considerable promise and could be used to create new treatments.

## Electronic supplementary material

Below is the link to the electronic supplementary material.

Supplementary Material 1

## Data Availability

The datasets used and analyzed during the current study are available from the corresponding author on reasonable request.

## Abbreviations

Ts: Trichinella spiralis
Tp: Trichinella pseudospiralis
RCL: Reactive center loop
Serpins: Serine protease inhibitors
