# Supplementary material for: Comparison of structures and inhibition activities of serine protease inhibitors of Trichinella spiralis and Trichinella pseudospiralis
Source: Cell Biosci. 2025 Mar 13;15:35. doi: 10.1186/s13578-025-01375-0 (PMC11905679; doi:10.1186/s13578-025-01375-0)
Supplement: Supplementary file 1 — Supplementary Material 1 [file 13578_2025_1375_MOESM1_ESM.docx]

Supporting Information for

**Comparison of structures and** **inhibition activities of serine protease inhibitors of *Trichinella spiralis* and *Trichinella pseudospiralis***

Figure S1. Comparsion of structure of Tp-serpin, Ts-serpin with Other Serpin
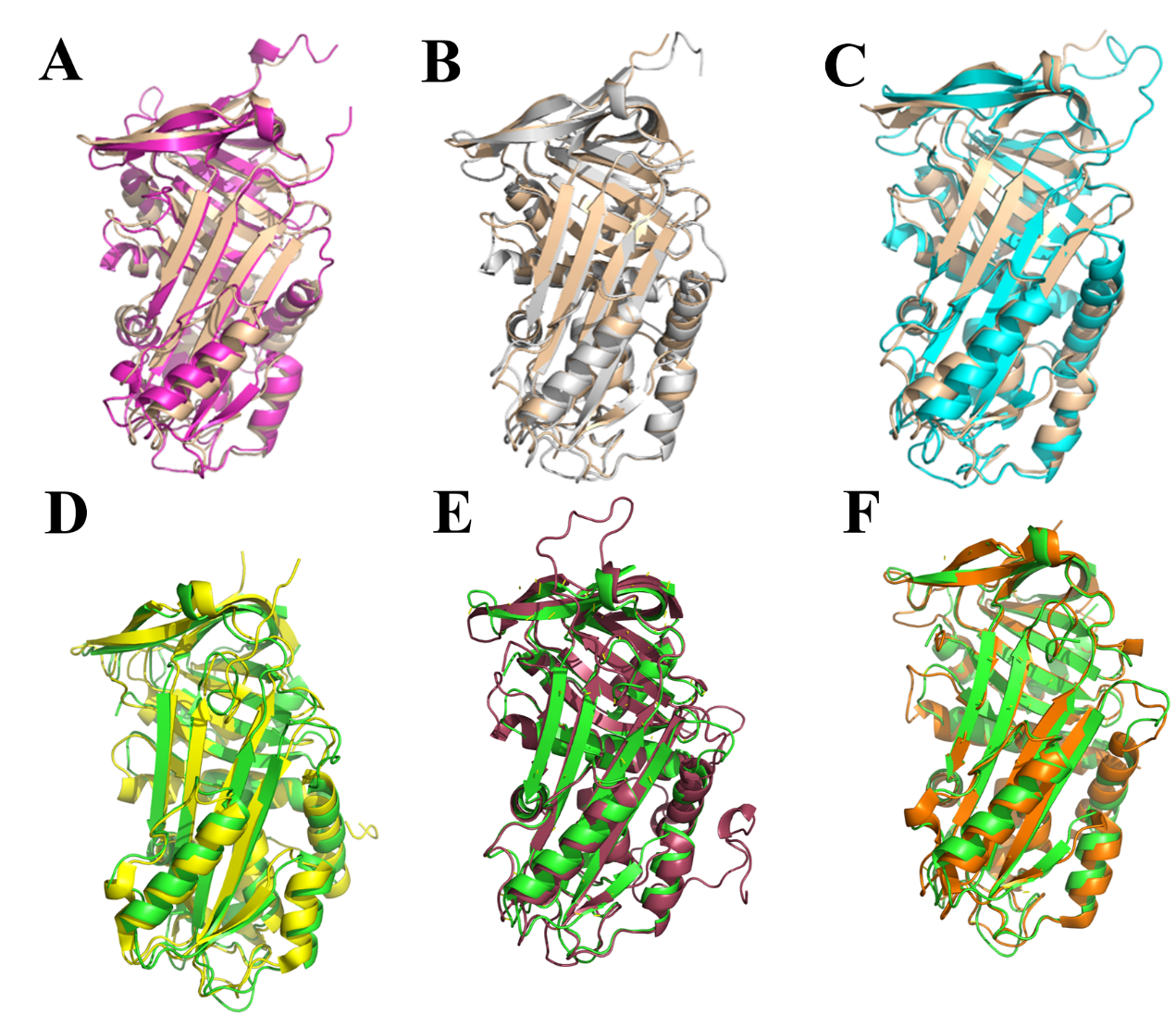
s.

(A) (B) (C) Comparsion of structure of Tp-serpin in golden with human Neuroserpin (PDB:3FGQ) in magenta, SERPINB1 (PDB:4GA7) in magenta, Maspin (PDB:1WZ9) in cyan. (D) (E) (F) Comparsion of structure of Ts-serpin in green with Antithrombin (PDB:2BEH) in yellow, MENT (PDB:2H4R) in chocolate, serpin SPN48 (PDB:3OZQ) in orange.

Table S1. The information of similar structure of Tp-serpin

| Number | Z-value | RMSD（Å） | Similarity (%) | PDB | Name |
| --- | --- | --- | --- | --- | --- |
| 1 | 44.5 | 1.6 | 34 | 4GA7 | Leukocyte elastase inhibitor |
| 2 | 44.3 | 1.6 | 26 | 1WZ9 | MASPIN |
| 3 | 43.5 | 1.9 | 30 | 1UHG | Ovalbumin |
| 4 | 42.7 | 1.7 | 36 | 2ZV6 | SERPIN B3 |
| 5 | 42.4 | 1.9 | 33 | 2H4R | MENT |
| 6 | 42.4 | 1.9 | 24 | 4DTE | SERPIN E |
| 7 | 42.2 | 1.9 | 31 | 3Q02 | Plasminogen activator inhibitor |
| 8 | 42.1 | 2.2 | 33 | 3KCG | Clotting factor IX A light chain |
| 9 | 42.1 | 2.0 | 28 | 1SEK | SERPIN K |
| 10 | 42.0 | 2.1 | 33 | 1SR5 | Antithrombase Ⅲ |
| 11 | 42.0 | 1.9 | 32 | 1AZX | Antithrombase |
| 12 | 41.7 | 1.9 | 38 | 5CDX | CONSERPIN |
| 13 | 41.7 | 2.1 | 31 | 4DY0 | GLIA Derived tubulin |
| 14 | 41.6 | 2.0 | 34 | 2GD4 | Blood coagulation factor Ⅹ |
| 15 | 41.5 | 1.8 | 33 | 1BY7 | Plasminogen activator inhibitor-2 |
| 16 | 41.3 | 1.9 | 28 | 3LE2 | SERPIN-ZX |
| 17 | 41.1 | 2.3 | 32 | 3FGQ | α1-Antitryptase |
| 18 | 41.1 | 1.8 | 27 | 6CJ7 | SERPIN-12 |
| 19 | 40.8 | 2.0 | 28 | 4RO9 | SERPIN-2 |
| 20 | 37.4 | 3.1 | 26 | 4AU2 | SERPIN H |


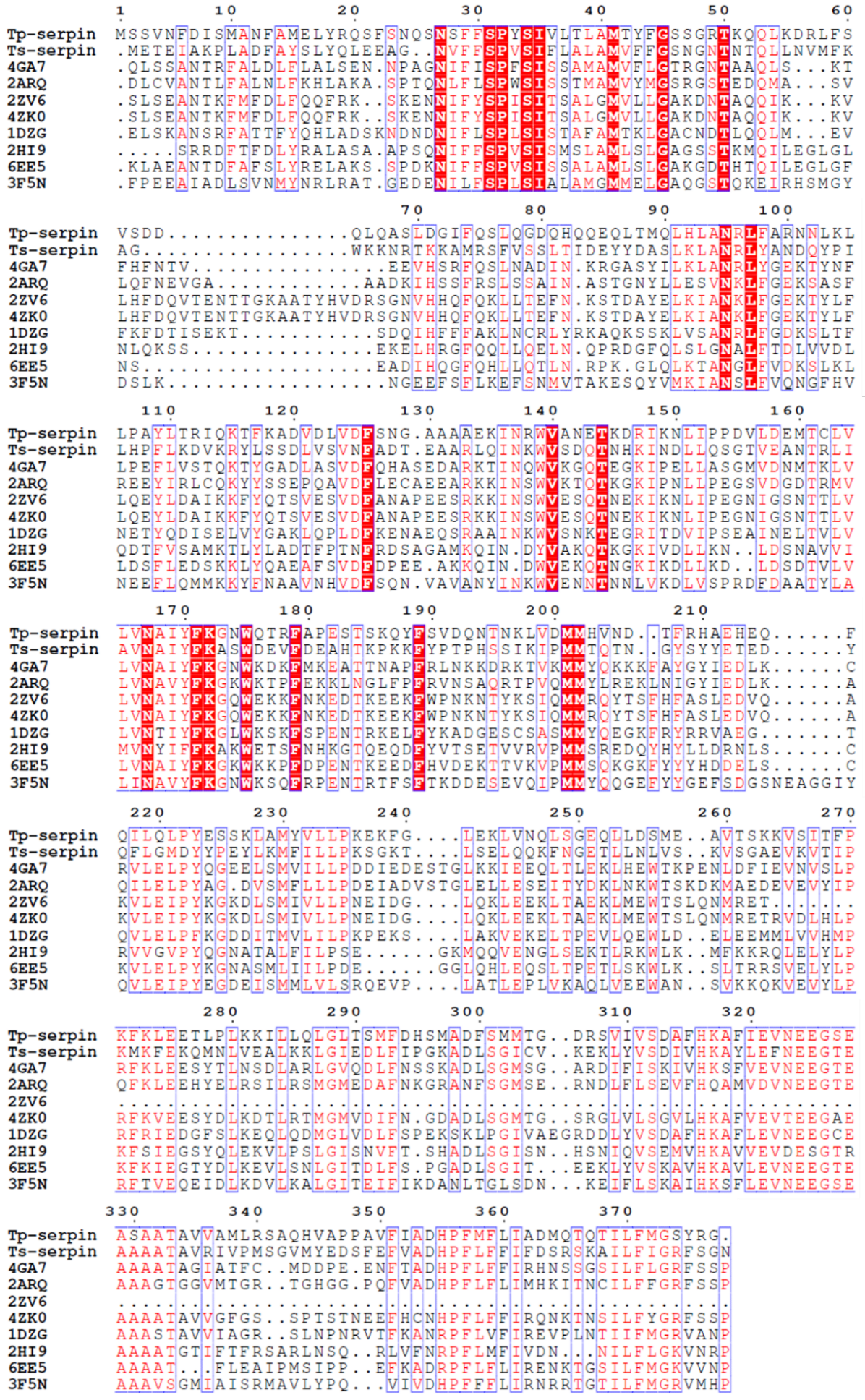
Figure S2. Sequences alignment of Tp-serpin and Ts-serpin with similar structures

Table S2. The information of similar structure of Ts-serpin

| Number | Z-value | RMSD（Å） | Similarity (%) | PDB | Name |
| --- | --- | --- | --- | --- | --- |
| 1 | 46.2 | 1.6 | 33 | 2BEH | Antithrombase |
| 2 | 45.8 | 1.5 | 32 | 2ANT | Antithrombase Ⅲ |
| 3 | 45.4 | 1.6 | 33 | 2H4R | MENT |
| 4 | 44.2 | 1.7 | 39 | 4GA7 | Leukocyte elastase inhibitor |
| 5 | 43.8 | 1.9 | 34 | 2ZV6 | SERPIN B3 |
| 6 | 43.5 | 1.7 | 30 | 6HGE | α- Antichymotrypsin |
| 7 | 43.5 | 2.0 | 30 | 4X30 | Thyroid binding globulin |
| 8 | 43.0 | 1.9 | 29 | 4DY0 | GLIA Derived tubulin |
| 9 | 42.6 | 2.0 | 31 | 6F02 | Human kallikrein binding protein |
| 10 | 42.6 | 1.8 | 28 | 1JMJ | Heparin cofactor |
| 11 | 42.6 | 1.7 | 35 | 4ZK0 | SERPIN B4 |
| 12 | 42.2 | 2.0 | 28 | 3Q03 | Plasminogen activator inhibitor |
| 13 | 41.8 | 2.1 | 34 | 1OVA | Ovalbumin |
| 14 | 41.8 | 2.4 | 29 | 1YXA | SERPIN E |
| 15 | 41.4 | 2.3 | 31 | 3F5N | Neurogenic serine protease inhibitors |
| 16 | 41.3 | 1.9 | 42 | 6EE5 | CONSERPIN |
| 17 | 41.2 | 2.3 | 32 | 1WZ9 | Clotting factor IX A light chain |
| 18 | 41.2 | 2.0 | 30 | 5IO1 | α1- Antitryptase |
| 19 | 40.9 | 2.1 | 25 | 3F1S | Protein Z-dependent protease inhibitors |
| 20 | 40.6 | 2.2 | 31 | 2GD4 | Blood coagulation factor Ⅹ |

Table S3. Hydrogen bonds and salt bridges of Tp-serpin

| Hydrogen bonds | | | | | |
| --- | --- | --- | --- | --- | --- |
| # | Structure 1 | | Dsit.[Å] | Structure 2 | |
| 1 | Asn | 137[ND2] | 3.10 | ILe | 152[N] |
| 2 | Phe | 98[N] | 2.88 | Val | 164[O] |
| 3 | Asn | 138[OD1] | 2.81 | Ile | 152[O] |
| 4 | Phe | 98[O] | 2.94 | Val | 164[N] |
| 5 | Asn | 137[O] | 3.32 | Ile | 148[O] |
| 6 | Val | 140[O] | 2.87 | Thr | 144[OG1] |
| 7 | Val | 140[O] | 2.96 | Thr | 144[N] |
| 8 | Ala | 141[O] | 2.62 | Asp | 146[N] |
| 9 | Gln | 90[O] | 2.77 | Lys | 172[N] |
| 10 | Gln | 90[N] | 2.65 | Lys | 172[O] |
| 19 | Arg | 96[O] | 2.99 | Val | 166[N] |
| 12 | His | 92[O] | 2.96 | Tyr | 170[N] |
| 13 | His | 92[N] | 2.93 | Tyr | 170[O] |
| 14 | Arg | 100[N] | 3.14 | Cys | 162[O] |
| 15 | Ala | 94[O] | 2.97 | Ala | 168[N] |
| 16 | Ala | 94[N] | 2.90 | Ala | 168[O] |
| 17 | Arg | 96[N] | 3.09 | Val | 166[O] |
| Salt bridges | | | | | |
| # | Structure 1 | | Dsit.[Å] | Structure 2 | |
| 1 | Asn | 137[ND2] | 3.20 | Asn | 150[OD1] |
| 2 | Ans | 137[O] | 2.74 | Ile | 151[N] |
| 3 | Gln | 90[NE2] | 2.75 | Tyr | 170[OH] |
| 4 | Asn | 95[OD1] | 2.84 | Asn | 167[ND2] |

Table S4. Hydrogen bonds and salt bridges of Ts-serpin

| Hydrogen bonds | | | | | |
| --- | --- | --- | --- | --- | --- |
| # | Structure 1 | | Dsit.[Å] | Structure 2 | |
| 1 | Arg | 91[O] | 3.04 | Val | 161[N] |
| 2 | Leu | 147[O] | 3.07 | Arg | 128[NH1] |
| 3 | Tyr | 93[O] | 2.80 | Ile | 159[N] |
| 4 | Ile | 143[O] | 3.24 | Thr | 139[OG1] |
| 5 | Tyr | 93[N] | 2.67 | Ile | 159[O] |
| 6 | Lys | 143[O] | 3.24 | Thr | 139[OG1] |
| 7 | Lys | 142[N] | 2.93 | Thr | 139[O] |
| 8 | Arg | 91[N] | 3.08 | Val | 161[O] |
| 9 | Asn | 144[OD1] | 2.52 | Ser | 136[OG] |
| 10 | His | 141[N] | 2.68 | Ser | 136[O] |
| 11 | Ala | 89[N] | 2.81 | Ala | 163[O] |
| 12 | Ala | 89[O] | 2.94 | Ala | 163[N] |
| 13 | Asp | 83[O] | 2.19 | Lys | 167[NZ] |
| 14 | Asn | 95[N] | 2.89 | Arg | 157[O] |
| 15 | Ser | 85[O] | 2.86 | Lys | 167[N] |
| 16 | Ser | 85[N] | 2.66 | Lys | 167[O] |
| 17 | Lys | 87[N] | 2.83 | Tyr | 165[O] |
| 18 | Lys | 87[O] | 2.76 | Tyr | 165[N] |
| Salt bridges | | | | | |
| # | Structure 1 | | Dsit.[Å] | Structure 2 | |
| 1 | Leu | 147[O] | 2.78 | Asn | 132[ND2] |
| 2 | Leu | 147[N] | 2.69 | Asn | 132[OD1] |
| 3 | Ile | 143[N] | 3.07 | Thr | 139[OG1] |
| 4 | His | 141[NE2] | 2.60 | Asp | 137[OD1] |
| 5 | Asp | 83[OD2] | 2.20 | Lys | 167[NZ] |


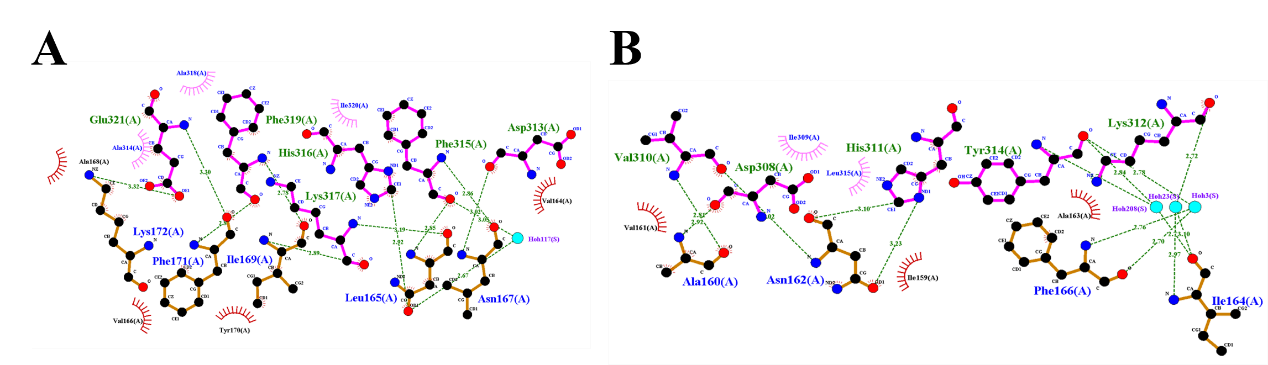
Figure S3. Structural characteristics of Tp-serpin and Ts-serpin

(A) The interaction between s3A and s5A of Tp-serpin was analyzed by LigPlot^+^. The amino acids of s5A in green, the amino acids of s3A in blue, the sky blue ball represents the water molecules, and dashed lines represent hydrogen bonds. (B) The interaction between s3A and s5A of Ts-serpin was analyzed by LigPlot^+^. The amino acids of s5A in green, the amino acids of s3A in blue, the sky blue ball represents the water molecules, and dashed lines represent hydrogen bonds.


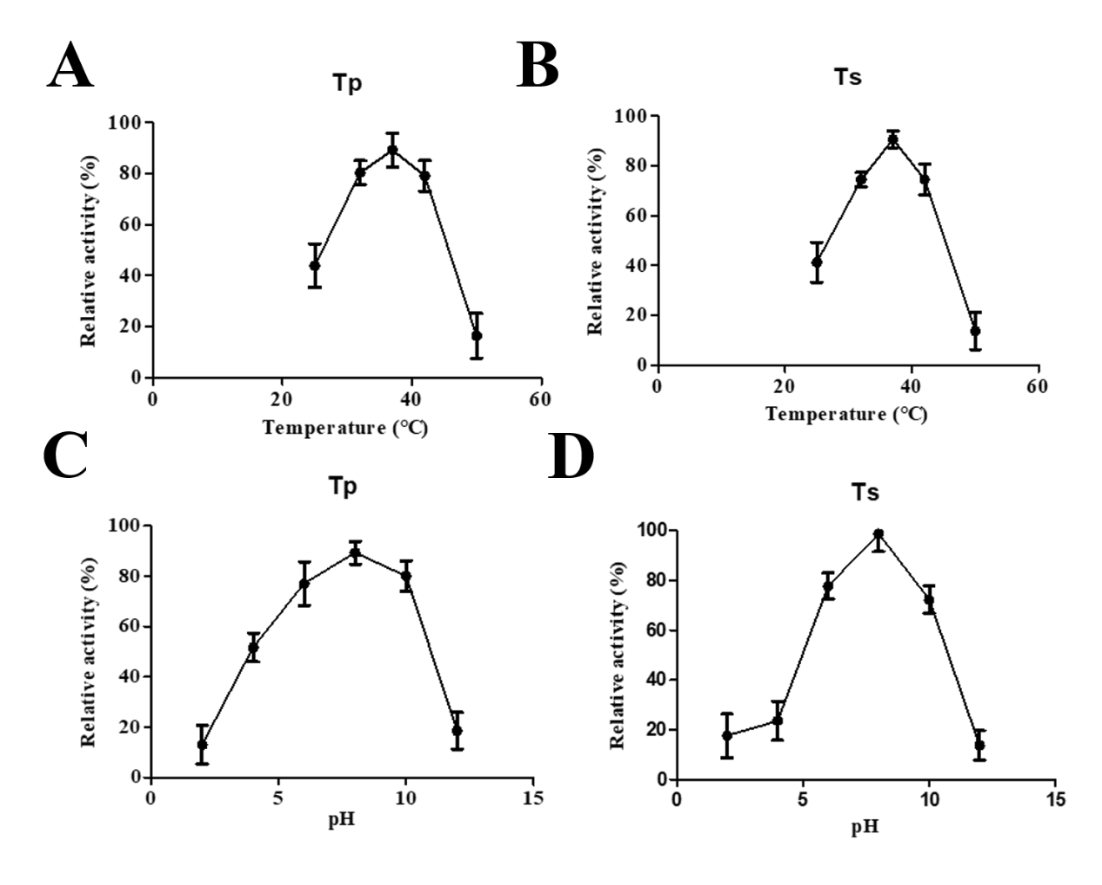


Figure S4. Temperature-dependent and pH -dependent inhibitory assays

(A) (B) Enzyme inhibition activity of serpin in different temperature. (C) (D) Enzyme inhibition activity of serpin in different pH.
